# Cooking Frequency and Perception of Diet among US Adults Are Associated with US Healthy and Healthy Mediterranean-Style Dietary Related Classes: A Latent Class Profile Analysis

**DOI:** 10.3390/nu12113268

**Published:** 2020-10-25

**Authors:** Nicole Farmer, Lena J. Lee, Tiffany M. Powell-Wiley, Gwenyth R. Wallen

**Affiliations:** 1 National Institutes of Health Clinical Center, Bethesda, MD 20814, USA; jumin.park@nih.gov (L.J.L.); gwallen@cc.nih.gov (G.R.W.); 2 Social Determinants of Obesity and Cardiovascular Risk Laboratory, Cardiovascular Branch, Division of Intramural Research, National Heart, Lung, and Blood Institute, National Institutes of Health, Bethesda, MD 20892, USA; tiffany.powell-wiley@nih.gov; 3 Intramural Research Program, National Institute on Minority Health and Health Disparities, National Institutes of Health, Bethesda, MD 20892, USA

**Keywords:** dietary patterns, cooking frequency, perceived diet quality, Healthy Eating Index

## Abstract

Background: Meal habits are associated with overall dietary quality and favorable dietary patterns determined by the Healthy Eating Index (HEI). However, within dietary patterns, complexities of food combinations that are not apparent through composite score determination may occur. Also, explorations of these food combinations with cooking and perceived diet quality (PDQ) remain unknown. Methods: Data from the National Health and Nutrition Examination Survey (NHANES) 2007–2010 were utilized to determine the frequency of cooking at home and PDQ, along with sociodemographic variables. Latent class profile analysis was performed to determine person-centered data-driven analysis using the dietary index, HEI-2010, at both the daily and dinner meal-time levels. Multinomial logistic regression analysis was utilized to evaluate the association of dietary patterns with all covariates. Results: For daily HEI, five distinct dietary classes were identified. For dinner HEI, six classes were identified. In comparison to the standard American diet classes, home cooking was positively associated with daily (*p* < 0.05) and dinner (*p *< 0.001) dietary classes that had the highest amounts of total vegetable and greens/beans intake. PDQ was positively associated with these classes at the daily level (*p* < 0.001), but negatively associated with healthier classes at the dinner level (*p* < 0.001). Conclusion: The use of latent class profile analysis at the daily and dinner meal-time levels identified that food choices coalesce into diverse intakes, as shown by identified dietary classes. Home cooking frequency could be considered a positive factor associated with higher vegetable intake, particularly greens/beans, at the daily and dinner levels. At the same time, the perception of diet quality has a positive association only with daily choices.

## 1. Introduction

Evidence suggests that overall dietary patterns, rather than nutrient- or food type-specific analysis, may best reflect the complexity of food consumption and its relation to dietary behavior and quality. Dietary patterns may be especially important when considering how to apply food consumption to person-centered dietary counseling to encourage adherence to recommended guidelines [1,2,3,4,5,6]. Dietary patterns may also help to evaluate intrapersonal and social determinants that influence food choice and diet quality: environmental and neighborhood access to food [7]; socioeconomic factors [8]; gender [9,10]; and psychosocial factors, including perceived diet quality [11]. 

Meal habits, such as home cooking, are factors in food consumption that reflect daily life structure [12], social context and determinants [13], and intrapersonal factors [14]. All of these factors may influence food choice and selection at meal time and on a daily basis. Thus, it is logical that cooking is reflective of or associated with the consumption of health-promoting dietary patterns, such as patterns consistent with the Healthy Eating Index [15], the Dietary Action to Stop Hypertension (DASH) diet and the Mediterranean diet [16]. The association of home cooking with dietary patterns likely represents a bidirectional system, in which those who have higher usage of health-promoting diets will cook frequently to maintain that diet. However, even within a dietary pattern, consistent concordance to a particular dietary regimen or food groupings may not readily occur. For instance, the decision to eat leafy greens may be juxtaposed with the decision to eat refined starch foods within one day or within a meal. Hence, to understand the overall impact on dietary quality for home cooking, it is important to ascertain food groupings that may occur together daily that could influence overall diet quality. Meal-specific analysis of dietary patterns are additionally significant to consider in parallel analysis to daily in order to study diet-disease and diet-behavior patterns, as diet behaviors that effect food choice may differ by meal [17]. Thus, meal-specific analysis provides an approach to also deal with the complexity and potential irregularity of diet intake. 

Despite the growing interest in dietary patterns, challenges in accurate determination of dietary patterns still exist, primarily because of the need for consistent strategies to reduce the complex multidimensional nutritional data down to an interpretable set of observed patterns [17]. This reduction in complex dietary data into dietary patterns can be achieved through either a hypothesis-oriented approach or a statistical approach. The hypothesis-driven approach uses previous dietary information to stratify a dietary pattern. The Healthy Eating Index is an example. Data analysis of hypothesis-driven dietary patterns usually includes variable-oriented analysis, which focus on relationships among variables (e.g., regression, factor analysis, and structural equation modeling) [18,19,20]. However, variable-oriented analyses limit translation of findings to individuals since the information obtained by this particular statistical method is variable oriented, not individual oriented [21]. A statistical approach to developing a dietary pattern involves taking specific data to rank, cluster or group individuals and may be able to identify connections between foods or food groups contained in an array of nutritional data [22]. 

An individual-oriented approach, which focuses on identifying the relationship among individuals (e.g., cluster analysis and latent class analysis), may be preferred in nutrition and dietary pattern research. The goal of these approaches is to divide heterogeneous group units into homogenous subgroups, in which members are similar to each other while different from individuals in other subgroups [23]. Initial work by Guenther et al. at the National Cancer Institute (NCI) led to the first individual-level analysis of a hypothesis-based dietary pattern using the Healthy Eating Index 2010 (HEI-2010). This analysis used principal component analysis (PCA) to group data. However, PCA does not consider “unobserved” subgroup patterns. Latent class analysis may provide a superior approach as it considers unobserved (i.e., latent) groups, is amenable to different data types, creates mutually exclusive classes, and for population-based studies, may be modified for sampling or response bias. Latent class analysis takes into account the relationships between variables in identifying groups of people who are similar to each other, and thus may offer an advantage over univariate approaches. Latent class analysis also may show a realistic picture of what people eat in daily life [17]. Increasingly, empirical results from latent class analysis suggest a preference in comparison to cluster analysis and factor analysis [24,25]. However, only a few studies have used latent class analysis alone to further evaluate dietary patterns [17,25,26]. To date, none of these studies utilized the National Health and Nutrition Examination Survey (NHANES) data to establish dietary pattern clusters and identify predictors of dietary patterns. A large, nationally representative, population-based study such as the NHANES may provide a comprehensive and more generalizable picture of dietary patterns in US adult populations. Our study builds on the current literature and adds to the current discussion by focusing on meal habits and perceived diet quality. 

The aim of our analysis was to investigate dietary classes through latent class analysis using the HEI-2010 from a sample of NHANES 2007–2010 US adults. Our hypothesis is that, through the use of latent class analysis, we will identify unique dietary classes among the US population and that household cooking, sociodemographic variables, and higher perception of diet quality will be positively associated with healthier dietary classes. 

## 2. Materials and Methods 

### 2.1. Data and Sample

This study was a secondary data analysis (N = 12,472) obtained from the NHANES, a cross-sectional survey designed to monitor the health and nutritional status of the civilian non-institutionalized US population. The survey consists of interviews conducted in participants’ homes and standardized physical examinations, including measured height and weight, in mobile examination centers. The NHANES sample is selected through a complex, multistage probability design. Non-Hispanic black and Hispanic persons, among other groups are oversampled to obtain reliable estimates for these population subgroups [27]. The NHANES 2007–2010 sample data of adults (age 19 and older) were analyzed, producing estimates and standard errors for the NHANES sample population. To ensure the equal probability of being sampled, weightings were assigned to each subject. The selected data cycle of 2007–2010 is the last year where questions regarding cooking at home practices were used. In the daily dietary pattern analysis, 11,481 subjects were included after excluding 991 subjects with incomplete responses of the dependent variable, diet quality. In the dinner dietary pattern analysis, a total of 10,556 participants were included following exclusion of 1916 participants with incomplete responses of the diet quality. 

### 2.2. Measures

Objective Diet Quality: An objective measure of dietary quality was determined using the HEI-2010. The HEI-2010 is a 12-component measure of diet quality in terms of conformance to the dietary guidelines for Americans, which are the basis of nutrition policy for the United States government and the foundation of all federal nutrition guidance [28]. Key features of HEI-2010 are that diet quality is assessed from the perspectives of adequacy and moderation, the scoring standards are density based such that the relative mix of foods (episodic versus non-episodic foods) is evaluated; and the standards for the maximum scores are the easiest to achieve recommendations among those that vary by energy level, sex, and/or age. Nine of the 12 components measure the consumption of adequate amounts of total fruit, whole fruit, greens and beans, whole grains, seafood and plant proteins that are considered episodically consumed. Total vegetables, dairy, total protein foods, and fatty acids, refined grains, sodium and SoFAAS (solid fats, alcohol and added sugars), are considered non-episodically consumed [29]. The latter three components measure the moderate consumption of refined grains, sodium, and SoFAAS, with lower numerical scores indicating higher consumption. 

We used an index-based approach to determine dietary pattern for this analysis because use of indices addresses the complexity of diet, multicollinearity between dietary components, and can be readily translated into dietary recommendations [5]. For both the NHANES 2007–2008 and 2009–2010 cycles, trained interviewers conducted the 24 h dietary recalls in person at the mobile examination center using the US Department of Agriculture Automated Multiple-Pass Methodology [30]. Day 1 dietary recall data were used because the majority of participants completed day 1 testing during the examination at the mobile examination center. Only dietary data determined reliable by interviewers were included in the NHANES database. To account for differences in the day of the week for dietary recall data, sample weights are calculated and applied to the diary data by the NHANES. The Simple HEI Scoring Algorithm Per Day code uses the NHANES 2007–2010 data, and calculates HEI-2010 scores using a simple method that does not account for measurement error. This program calculates HEI-2010 component and total scores for individuals, using data collected over at least two 24 h periods. This code creates unweighted HEI-2010 scores. Scores for total HEI and all components were analyzed as continuous variables. In addition to HEI, total daily kcal were calculated from the two 24 h diet recall entries. For the purpose of the latent class analysis approach, HEI components were examined for similarity of expected food types; if components represented similar food types, then the two components were combined to avoid redundancy. After similarity analysis, whole fruit was excluded to avoid overlapping with total fruit. Table 1 shows the 11 HEI-2010 components used in our analysis. Cooking frequency, the meal habit of interest for this analysis, was measured by the NHANES question that is meal specific for dinner. Dinner HEI-2010 component scores were determined by selecting dinner foods and beverages from the 24 h recall dietary records as described by Farmer et al. [14]. 

Demographic and socioeconomic covariates: Demographic and socioeconomic status variables (age, sex/gender, race/ethnicity, marital status, education level, employment status, poverty/income ratio, and total number of people in household) were defined using self-reported data. Marital status and education were calculated using NHANES variables, and after analysis of data frequencies, two categories were created for marital status (i.e., married/cohabitating, previously or never married) and three categories were created for education (i.e., ≤high school graduate or equivalent, some college, college/graduate and above). Employment status was calculated using NHANES occupation variables in relation to the prior week: ‘working at a job or business’ and ‘with a job or business but not at work’, ‘looking for work’ or ‘not working’. Variables were recoded to create a dichotomous employment status: not working in the prior week (including unemployed, retirees, those not actively looking for work, and those employed but nor working the prior week), and those working in the prior week. Poverty income ratio is the ratio of income to the federal poverty threshold based on family composition and size, and this parameter was used to develop cut offs for three income subgroups: lowest (≤130% of the poverty threshold), middle (131%–185%) and highest (>185%) [11]. 

Cooking frequency covariate: Cooking frequency was evaluated using the NHANES question “In the past 7 days, how many times did you or someone in your household cook dinner at home?” Analysis was conducted using the response as a continuous variable with calculation of means. Maximum number of responses for the answer based on weekly data was seven, and thus responses were excluded which provided an answer > 7. Responses were categorized in order to allow for practical interpretation and to maintain consistency with the current literature [14,31]. Data were analyzed to ensure that these categories were robust for our sample population. Three categories were used for this analysis based on the number of dinners cooked per week: 0–2 (‘low’), 3–5 (‘moderate’) and 6–7 (‘high’).

Perceived diet quality covariate: Perceived diet quality (PDQ) was determined using the NHANES Diet Behavior and Nutrition questionnaire (DBQ), ‘In general, how healthy is your overall diet?’ on a 5-point Likert scale, with possible answers ranging from ‘excellent’ to ‘poor’. Participants’ responses to this question were used to generate PDQ scores. Perceived diet quality was scored as ‘high’ for those who perceived their diet to be ‘excellent’ or ‘very good’, ‘medium’ for those who perceived their diet to be ‘good’ and ‘low for those who perceived their diet to be ‘fair’ or ‘poor’ [11]. 

### 2.3. Statistical Analysis

As described previously, latent class analysis, a type of finite mixture model, was used to identify homogenous unobserved subgroups (i.e., latent classes) that exist within a heterogeneous population using multiple observed indicators. The fundamental latent class model postulates that there are unobservable subgroups, which are called classes, within a population [32] and identifies meaningful unobserved subgroups (i.e., latent classes) based on similarities in responses to a set of observed indicators [33]. Latent class analysis typically uses categorical variables. When continuous variables are analyzed, latent class analysis is called latent class profile analysis (LCPA) [33]. 

**Figure 1:**
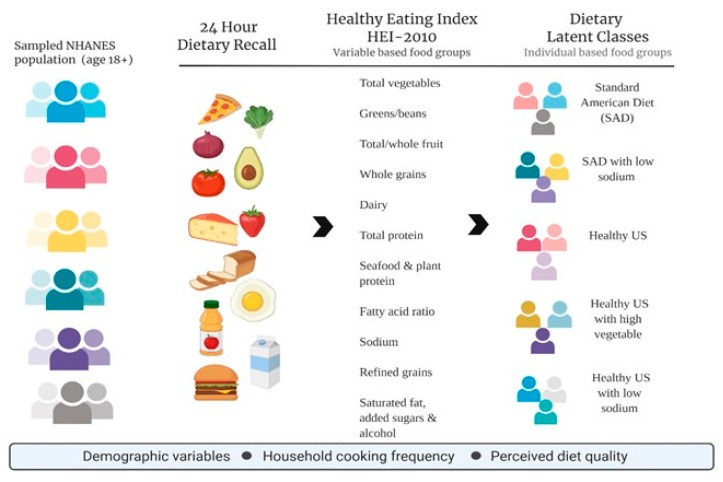
Overview of latent class profile analysis (LCPA) using daily Healthy Eating Index-2010 (HEI-2010) from dietary recall data collected from sampled National Health and Nutrition Examination Survey (NHANES) adults (age 18+).

In this study, LCPA was used to identify the underlying dietary classes (i.e., latent classes) based on responses to 11 HEI-2010 diet components (Figure 1). To determine whether the use of weights within the LCPA was needed, a sensitivity analysis was performed. In this study, an unweighted LCPA analysis was conducted because parameter estimates were substantively analogous with and without weighting [34]. Estimation was carried out with robust maximum-likelihood and the expectation-maximization algorithm [35]. Several statistical fit indices were used to assess model fit and to determine the optimal number of latent classes: the Akaike information criterion (AIC) [36], the Bayesian information criterion (BIC) [37], the Vuong–Lo–Mendell–Rubin likelihood ratio test (VLMR), the parametric bootstrapped likelihood ratio test (BLRT), and entropy [38]. The model that fits the data best has the lowest BIC and a VLMR and/or BLRT, which indicates that the estimated model is a better fit than the model with one fewer class and higher entropy value [39]. Mplus version 7.2 [40] was used for this analysis.

Once latent classes were identified, multinomial logistic regression was used to determine whether demographic and socioeconomic factors, cooking frequency and PDQ predicted inclusion within a class. In determining whether to use weighted data in a regression model, use of the weights (weighted ordinary least square, WOLS) showed unbiased and consistent parameter estimates, but unweighted ordinary least square (OLS) provided unbiased and consistent estimates with smaller standard errors in this study. Consistent with recommendations from the literature, unweighted models were then used [41,42]. Well-established demographic and socioeconomic risk factors for dietary behaviors such as age, sex/gender, and marital status were entered in all models [13]. Variables with a significant relationship in univariate analyses were retained in multivariate analyses of predicted inclusion in a class. The statistical analyses were performed with SPSS version 25.0 [43]. 

## 3. Results

### 3.1. Demographic and Socioeconomic Characteristics and Dietary Habits of Study Population 

Table 2 shows sample-weighted demographic and socioeconomic characteristics and dietary habits for the NHANES 2007–2010 participants. The mean age of the study population was 46.32 years and 52.2% were female. Concerning race and ethnicity, 69.1% of the sample identified as Non-Hispanic White (NHW), 13.6% as Mexican American (MA) or Other Hispanic (OH), 11.5% as Non-Hispanic Black (NHB), and 5.8% as Other (O). In the sample, 62.5% were married or living with a partner, 26.1% had the highest level of education obtainment, 66.3% were in the highest poverty-to-income ratio tertile. Regarding cooking and dietary perception, the mean frequency of dinners cooked per week within an average household was 3.03 and 31.8% of the population reported high self-perception of their diet. 

### 3.2. Identification, Labeling and Description of Dietary Classes

By using LCPA, identification of five distinct classes occurred for daily HEI, and six classes for dinner HEI through the use of several statistical fit indices (Table 3). Within daily HEI and dinner HEI, there were internal groups of classes that diverged by intake patterns (Supplement Tables S1 and S2, respectively). Healthy Eating Index component scores of each class were compared to reported US averages for adults for HEI-2010, as reported by Guenther et al. [29]. For this grouping, daily intakes were used for the classification of dinner classes. 

**Figure 2:**
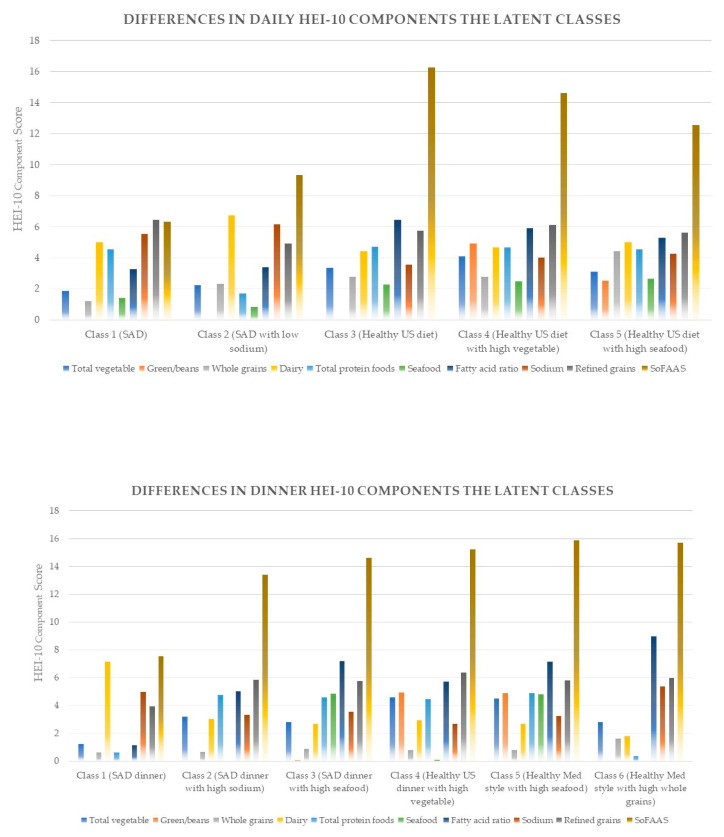
Latent classes representing differences in the daily and dinner HEI-2010 components of the NHANES 2007–2010 sample population (age ≥ 19 years).

For the identified classes, the naming occurred in two steps. First, we identified those classes consistent with average intakes of each food component score (Supplement Tables S1 and S2). Second, we utilized current literature regarding dietary patterns. Those consistent with standard American diet (SAD) patterns were determined by the presence of empty calories and refined grains [44]. The other classes were determined to be consistent with the 2015–2020 US dietary guidelines for healthy US-style pattern (vegetables, fruits, grains, dairy, protein) or the healthy Mediterranean (Med)-style (higher vegetable, fruit, seafood, and less dairy than healthy US style) pattern [45]. Both the healthy US-style pattern and the healthy Med-style pattern do not consider sodium in their defining food groups. When classes were determined to have a differing sodium intake, we identified them as SAD, healthy US style or healthy Med-style plus the level of sodium intake. Table 4 shows a listing of all classes. Figure 2 shows differences in the daily and dinner HEI-2010 components for identified latent classes. 

As shown in Table 5, Class 1 (*n* = 2908, 25%), labeled “SAD”, was not only characterized by SoFAAS and refined grains, but also low total fruit and whole grains. Class 2 (*n* = 1568, 14%) was characterized in relation to SAD but was found to have lower sodium, total protein and seafood and was thus labeled “SAD with low sodium.” Class 3 (*n*= 3798, 33%), labeled “healthy US”, was characterized by higher sodium, higher fatty acid and higher vegetable intake than SAD, but lower SoFAAS intake. Class 4 (*n* = 2502, 22%), labeled “healthy US with high vegetable”, was characterized by highest vegetable and greens/beans intake of all the class. Class 5 (*n* = 705, 6%) was characterized by lower sodium intake than the healthy US diet (Class 3), and was labeled “healthy US with low sodium”. 

As shown in Table 6 for the dinner classes, Class 1 (*n* = 1431, 14%), labeled “SAD dinner”, was characterized by the aforementioned SAD criteria and low total fruit and whole grains and refined grains at dinner compared to other dinner classes. Class 2 (*n* = 5028, 48%) was characterized by SAD dinner characteristics with higher sodium and was labeled “SAD dinner with high sodium.” Class 3 (*n* = 1380, 13%), labeled “SAD dinner with high seafood”, was characterized by similar characteristics of Class 2 but with higher seafood. Class 4 (*n* = 1396, 13%) was characterized by high vegetable, greens/beans, high fatty acid, and low refined grains and was labeled “healthy US dinner with high vegetable.” Class 5 (*n* = 737, 7%), labeled “healthy Mediterranean-style dinner”, was characterized by the same characteristics as Class 4 except with a high seafood intake. Class 6 (*n* = 584, 6%) was characterized by lower vegetable intake, lower sodium intake and higher whole grains and was such labeled as “healthy US dinner with low sodium”. 

Table 5 and Table 6 show differences in mean HEI component scores and energy (kcal) across all latent classes. All HEI-2010 components and energy (kcal) varied significantly across all five and six classes, respectively. 

### 3.3. Sociodemographic and Meal Preparation Habits Variables and Daily HEI Dietary Classes

Gender differences by Daily HEI dietary classes were identified. Females were more frequently in the class SAD with low sodium, while men were more frequently in the class SAD. Women were more frequent in all daily healthy US classes (Supplement Table S3). 

Percentages among the dietary classes for Mexican Americans and Other Hispanics were determined not to be different (See Table 7). Thus, these two race/ethnicity classes were combined for further logistic analysis to represent one category: Mexican American/Other Hispanic (MA/OH). Unadjusted models showed significant differences across daily dietary classes for race/ethnicity, education, income, the number of people in household, the frequency of cooked dinner at home, and PDQ. Thus, these variables were retained in the adjusted model. Table 7 displays multinomial logistic regression results, with predictors for each class, using SAD (Class 1) as the reference. 

Class 2 (SAD with low sodium) versus Class 1 (SAD). Race/ethnicity (being MA/OH, or Other more likely and being NHB less likely compared to NHW) significantly predicted the likelihood of being in the SAD with low sodium group (Class 2) compared with the SAD (Class 1). 

Class 3 (healthy US) versus Class 1. Race/ethnicity (being MA/OH or Other more likely compared to NHW), education level, income, and PDQ significantly predicted the likelihood of being in the healthy US (Class 3) group compared with the SAD (Class 1). People with high or medium levels of PDQ had a higher likelihood of membership in Class 3 versus Class 1 than those with low levels of PDQ (odds ration [OR] = 1.92; 95% confidence interval [CI] = [1.62, 2.27], OR = 1.43; 95% CI = [1.24, 1.67], respectively). 

Class 4 (healthy US with high vegetable) versus Class 1. Race/ethnicity (being MA/OH or Other more likely compared to NHW), education level, the frequency of cooked dinner at home, and PDQ significantly predicted the likelihood of being in the healthy US diet with high vegetable (Class 4) group compared with the SAD (Class 1). People with a high frequency of cooked dinner at home (6–7 per week) were 1.47 times (95% CI = [1.09, 1.98]) more likely to be in Class 4 versus Class 1 than those with a low frequency of cooked dinner at home (0–2 per week). People with high or medium levels of PDQ had a higher likelihood of membership in Class 4 versus Class 1 than those with low levels of PDQ (OR = 2.36; CI = [1.96, 2.85], OR = 1.44; CI = [1.22, 1.70], respectively). 

Class 5 (healthy US with low sodium) versus Class 1. Race/ethnicity (being MA/OH or Other more likely compared to NHW), and education level significantly predicted the likelihood of being in the healthy US with low sodium (Class 5) group compared with the SAD (Class 1).

### 3.4. Sociodemographic and Meal Preparation Habits Variables and Dinner HEI Dietary Classes

Gender differences by dinner HEI dietary classes showed that females were more frequently in all dinner classes, except for SAD dinner with high sodium (Supplement Table S3). 

Unadjusted models showed significant differences across dinner dietary classes for race/ethnicity, education, income, employment status, the number of people in household, the frequency of cooked dinner at home, and PDQ. Thus, these variables were retained in the adjusted model. Table 8 displays multinomial logistic regression results with predictors for each class, using SAD dinner (Class 1) as the reference. 

Class 2 (SAD dinner with high sodium) versus Class 1 (SAD dinner). Race/ethnicity (being MA/OH less likely and being NHB more likely compared to NHW) and the frequency of cooked dinner at home significantly predicted the likelihood of being in the SAD dinner with high sodium group (Class 2) compared with the SAD dinner (Class 1). People with a high frequency of cooked dinner at home (6–7 per week) were 1.23 times (95% CI = [1.00, 1.50]) more likely to be in Class 2 versus Class 1 than those with a low frequency of cooked dinner at home (0–2 per week). 

Class 3 (SAD dinner with high seafood) versus Class 1. Race/ethnicity (being NHB or Other more likely compared to NHW), education level, and income significantly predicted the likelihood of being in the Class 3 group compared with the SAD dinner (Class 1). 

Class 4 (healthy US dinner with high vegetable) versus Class 1. Race/ethnicity (being MA/OH or Other more likely compared to NHW), income, the frequency of cooked dinner at home, and PDQ significantly predicted the likelihood of being in the healthy US dinner with high vegetable (Class 4) group compared with the SAD dinner (Class 1). People with a high or moderate frequency of cooked dinner at home had a higher likelihood of membership in Class 4 versus Class 1 than those with a low frequency of cooked dinner at home (OR = 1.34; CI = [1.03, 1.74], OR = 1.41; CI = [1.08, 1.84], respectively). People with high levels of PDQ had a lower likelihood of membership in Class 4 versus Class 1 than those with low levels of PDQ (OR = 0.62; CI = [0.50, 0.78]). 

Class 5 (healthy Mediterranean-style dinner) versus Class 1. Race/ethnicity (being NHB or Other more likely compared to NHW), education level, income, the frequency of cooked dinner a home, and PDQ significantly predicted the likelihood of being in the Class 5 group compared with the SAD dinner class (Class 1). People with a high frequency of cooked dinner at home (6–7 per week) had a higher likelihood of membership in Class 5 versus Class 1 than those with a low frequency of cooked dinner at home (0–2 per week) (OR = 1.57; 95% CI = [1.13, 2.18]). People with high or medium levels of PDQ had a lower likelihood of membership in Class 5 versus Class 1 than those with low levels of PDQ (OR = 0.66; 95% CI = [0.51, 0.86], OR = 0.72; 95% CI = [0.57, 0.91], respectively).

Class 6 (healthy US dinner with low sodium) versus Class 1 (SAD). Race/ethnicity (being NHB or Other more likely compared to NHW) significantly predicted the likelihood of being in Class 6 compared with the SAD dinner class (Class 1). 

## 4. Discussion

In this analysis, we found dietary classes for daily intake and dinner from HEI-2010 food groups among a representative sample of US adults from the NHANES 2007–2010. Our findings make a unique contribution to the literature as it shows the presence of five daily dietary HEI-2010 classes and six dinner dietary HEI-2010 classes among American adults. To our knowledge, we are the first to report US adult dietary patterns using LCPA. The patterns derived from this LCPA provide a novel way to explore dietary behaviors, by identified unobserved (latent) population subgroups that are inferred observed variables. For example, our analysis identified nuanced categories within the SAD and healthy US diet, such as SAD with low sodium and healthy US with high vegetable, which emerged from the 24 h recall data. These nuanced categories represent the actual intake of individual adults, as opposed to determining what aspects of an adult diet fit into preconceived dietary pattern categories. Furthermore, the findings in this study demonstrate that LCPA can be used to discover statistically valid subsamples of dietary classes that might provide information on the role of demographic factors, meal preparation practices and PDQ on overall diet quality.

Our findings introduce insight regarding how people may actually meet intakes for some foods, but this effort might be offset by an unfavorable intake of other foods despite adhering to overall dietary guidelines. Sociological studies show that dietary decisions on a daily basis consist of diverse and contrasting foods reflecting complexities in the decision-making process for food choice that extends beyond health concerns [46]. Findings from our study support the tenant that among the daily and dinner HEI components, the most common classes among American adults (Class 3 daily and Class 2 dinner) consisted of sharp contrasts in food groups. For instance, Class 3 (healthy US) had the lowest intake of SoFAAS but the highest intake of sodium. 

### 4.1. Cooking Frequency and Dietary Classes

Our findings imply that cooking frequency has a positive association with the daily and dinner diet classes that are high in vegetable intake consistent with other literature [13,47]. This is not surprising as cooking is seen as a behavior necessary for the preparation and consumption of vegetables. Interestingly, cooking frequency in this analysis for both dinner and daily HEI was associated with classes that had lower SoFAAS and the highest intakes of greens/beans. Beans, in particular, are a food type associated with cardiovascular health and also meal planning, a component in the planning process for home cooking, even when controlling for sociodemographic factors [48]. Further, lowered consumption of added sugars, a component of SoFAAS, may be protective from adverse health outcomes [49,50]. 

Our analysis also presents data at an individual level representing how cooking frequency is related to diverse combinations of food on a daily or dinner basis. Although we found that cooking is positively associated with healthier classes of intake, such as healthy US with high vegetable compared to SAD. We also found that cooking frequency was associated with classes with higher sodium scores. Our findings therefore suggest a role for nutrition education or culinary education that includes promoting cooking frequency. The latter can help individuals identify flavorful substitutions for sodium [51] and the former can help individuals gain awareness of sodium usage and intake. It is important to note that there still may be an overall healthier intake with more frequent cooking as even the majority of the higher sodium intake classes associated with cooking in our analysis still had scores on average with US adult sodium scores [29]. 

People who cook frequently may be people who are motivated to engage in cooking behavior because of time perception, culture, and social norm influences. These influences are not necessarily decisions based on health or nutrition knowledge and could help to explain the discordant intakes associated with cooking in our analysis, such as higher sodium intake among classes associated with cooking. Although our analysis controlled for sociodemographic factors, there are possible influences of the food environment on food access or food insecurity that were not accounted for in our analysis. It is possible that variation in types of food consumed among people reflect making the most of what is available, and what is available may not always be completely concordant with an ideal diet. Lastly, our findings open for discussion the idea that groups recently identified as having lower comparative cooking frequency, such as NHB [52], could potentially be disadvantaged from a higher overall dietary quality through lower engagement in cooking. Prior analysis from our group identified that among NHB, higher cooking frequency was however associated with high total vegetable intake, which may include starchy vegetables like potatoes [14]. 

### 4.2. Perceived Diet Quality and Dietary Patterns 

Perception of diet quality is positively associated with a higher objective diet quality as determined by prior studies [11]. Having a higher PDQ in our analysis was related to the two healthiest daily dietary classes (Class 3 and 4); collectively, these groups include ideal caloric-based intakes for vegetables, protein, fruits, and whole grains. Prior reports have shown those who report high PDQ are more likely to have dark green vegetables and less sugar-sweetened beverages within their diet [53]. However, PDQ was also associated with classes with higher sodium intake, which was a finding similar to that of Powell-Wiley et al. [11]. Interestingly, when associated with dinner classes, higher PDQ was negatively associated with healthier dietary classes compared to SAD dinner. This different relationship between PDQ on a daily versus dinner basis may stem from available food choices for dinner despite PDQ status. For example, daily choices may represent foods that are concordant with PDQ, perhaps due to daily choices reflecting individual choice and dinner foods reflecting more household choices. 

To further explain our findings, there may be an association between a person’s perception of their usual intake and perception of their diet quality. Dietary reports, such as the 24 h recall used in this analysis, are in fact memory-based measurements and possibly represent a person’s perception of their usual intake [54]. Thus, there may be an inherent relationship between perception of diet quality and dietary pattern reported, such as was used in our analysis. However, if our results were due solely to this, we would also anticipate seeing the converse, a relationship with PDQ and the lower quality diet pattern. We suspect that our findings more likely represent a person’s perceived susceptibility to a health risk, for example, those with high PDQ recognize eating an unhealthy diet pattern is a health risk. Thus, there is likely a role of diet self-perception in avoiding a less healthy pattern and promoting healthier ones. 

### 4.3. Socioeconomic and Demographic Variables and Dietary Patterns

Previous studies have demonstrated a role for socioeconomic and demographic factors and dietary quality with men reportedly having poorer-quality diets than women and young adults poorer-quality diets than older adults [29]. Higher socioeconomic status is also associated with higher dietary quality [22,55,56]. In general, our results showed similar unsurprising findings. However, we did find an association between income status and dietary classes with dinner rather than with daily food selection. In fact, those with lower income were more likely to be associated with the SAD diet than with the healthy US classes compared to those with a higher income. This may reflect the role of food choices at dinner or represent a confounding relationship with food access that is only apparent with dinner food selections.

Racial and ethnicity differences in diet quality are well reported [57,58], with most of the findings in the literature involving differences between NHB and NHW. In our analysis, we found that NHB were the only racial/ethnic group when compared to NHW to be more likely in the SAD daily class than the SAD with lower sodium class. Dietary intake among NHB for fiber, fruits and vegetables are typically lower compared to other racial/ethnic groups [59]. Further, non-Hispanic Blacks tend to have a larger percentage of calories obtained from added sugars as well [60].

In contrast to differences between NHB and NHW, similarities with regard to dietary patterns between NHW and NHB have been reported in the literature. Kell et al. [61] using a representative US sample from the REGARDS study found NHW and NHB had similar adherence to various types of dietary patterns, but differed in the magnitude of adherence. We too found that overall at the daily level there were mostly similarities between dietary patterns of NHB and NHW, similar to findings from Kuczmarski et al. [62].

Despite similarities at the daily level, NHB were more likely to be in dietary classes other than SAD when compared to NHW at the dinner level. Although this finding appears in contrast to the abovementioned differences in diet quality, particular food characteristics of SAD dinner that may not meet general cultural food choices, such as a higher amount of dairy, may drive the differences. However, our data might also represent diversity in dietary quality among NHB that is not appreciated without the use of a latent variable analysis. This hypothesis is supported by work done by James [63], in which previously unreported dietary diversity among NHB men and women was determined through cluster analysis.

Our findings indicate that MA and OH shared similar dietary patterns and when compared to NHW, were more likely to fall within dietary classes different from NHW. The tendency towards a healthier diet intake among Hispanic individuals is well reported, especially with regard to ability to prepare meals with vegetables [64,65]. However, tendency towards healthier diet pattern may represent cultural choices moderated by years of acculturation, not measured in our analysis.

### 4.4. Strengths and Limitations

Our study analysis is unique for several reasons. We explored dietary classes of a representative US sample using a standardized measure of diet quality, HEI-2010, which allows us to interpret our results and findings in relation to US dietary guidelines, thus strengthening the implications of our analysis. Another strength of our analysis is our use of an advanced statistical method, LCPA.

Despite our strengths, our analysis is not without limitations. One, since one of our objectives was to link dietary patterns with cooking frequency; we were limited in our use of NHANES data to the 2007–2010 cycle. Thus, more current trends in meal habits are not reflected in the 2007–2010 cycle [66]. Two, we cannot distinguish from our analysis whether the food from the dietary recall data was cooked at home or not. Three, the question on cooking frequency used includes all forms of cooking and depends on a respondent’s perception of what meal preparation steps constitute cooking. This may have an impact on objective diet quality as food easily prepared may have lower overall dietary quality. Four, classification within one of the “healthy” labeled classes in our analysis does not mean that individuals in these classes reached ideal or optimal levels of intake. Lastly, our study used self-reported recall data. The use of memory based dietary data has been reported as potentially unreliable data [67]. However, self-reported dietary assessment methods from the NHANES are tested against objective standards for total energy expenditure and thus can be a useful measurement technique for many types of studies including assessments to identify potential health risks associated with a range of foods and eating patterns [68]. Furthermore, the sample population in our study is from the NHANES 2007–2010, a population which underwent repeat dietary recall [69], a technique used to improve validity of 24 h recall to person’s usual intake [70].

## 5. Conclusions

Our LCPA of a representative sample of US adults illustrates that by emphasizing diet classes within a priori dietary patterns, we can gain insight into the various ways that food choices coalesce into diverse intakes. The findings from our analysis suggest that Americans’ dietary intake varies with regard to daily choices and dinnertime choices. Further, home cooking frequency and perception of diet quality are related to higher vegetable intake and lower SoFAAS intake at both the daily and dinner levels. However, our findings present variable nutrition outcomes as classes also contained higher sodium intake and therefore a need for nutrition education and or culinary education to offer alternatives for sodium among dietary classes associated with cooking frequency and higher perceived diet quality. Overall, our study suggests that cooking frequency, as a dietary behavior, may be a potential area of focus for dietary quality promotion efforts directed at helping Americans adhere to recommended dietary guidelines.

## Figures and Tables

**Table 1:** HEI-2010 components used in the latent class analysis approach with description, type of component and maximum score [29].

	Component	Clarifying Description (Type of Component)	Range
1	Total vegetable	Total vegetable, not dark green, but including legumes (adequacy)	0–5
2	Greens/beans	Dark-green vegetables, beans, or peas (adequacy)	0–5
3	Total fruit	Includes all fruit and 100% fruit juice (adequacy)	0–5
4	Whole grains	Grain products that are 100% whole grains (adequacy)	0–10
5	Dairy	Includes all milk products, such as fluid milk, yogurt, and cheese, and fortified soy beverages (adequacy)	0–10
6	Total protein foods	Includes legumes not counted in Total veg or Greens/beans (adequacy)	0–5
7	Seafood and plant proteins	Includes legumes, seafood, nuts, seeds, and soy products (adequacy)	0–5
8	Fatty acid ratio	Ratio of poly-monounsaturated fatty acids to saturated fatty acids (adequacy)	0–10
9	Sodium	(moderation)	0–10
10	Refined grains	(moderation)	0–10
11	SoFAAS	Calories from solid fats, alcohol and added sugars, threshold for counting alcohol is > 13 g/1000 kcal (moderation)	0–20

Note. HEI-2010 = Healthy Eating Index 2010; SoFAAS = solid fats, alcohol, and added sugars.

**Table 2:** Demographic and socioeconomic characteristics and dietary habits of the NHANES 2007–2010 population (age ≥19 years, N = 11,481, weighted population = 442,232,688).

Variables	Category	Sample		Unweighted Sample		
		% US Population or Mean	SE	*n* (%) or Mean	SD	Range
**Demographic and Socioeconomic Characteristics**						
Age (years)		46.32	0.01	49.20	18.27	19–80
Gender	Female Male	52.2 47.8	0.00	5058 (51.0) 5623 (49.0)		
Race/ethnicity	Mexican American/Other Hispanic Non-Hispanic/Black Non-Hispanic/White Other—Including Multiracial	13.6 11.5 69.1 5.8	0.00	3288 (28.6) 2242 (19.5) 5419 (47.2) 532 (4.6)		
Marital status	Married/cohabitating Previously or never married	62.5 37.5	0.00	6680 (59.8) 4496 (40.0)		
Education level	≤ high school graduate or equivalent Some college College, graduate and above	43.6 30.3 26.1	0.00	5956 (53.3) 3022 (27.1) 2187 (19.6)		
Employment status	Working Not working ^a^	41.2 58.8	0.00	5954 (51.9) 5526 (48.1)		
Poverty income ratio	Low (≤130%) Middle (131–185%) High (>185%)	22.2 11.4 66.3		3397 (32.6) 1475 (12.8) 5558 (48.4)		
Total number of people in household		3.03	0.00	3.21	1.70	1–7
**Diet Perception and Meal Habits**						
Energy (kcal/day)		2128.61	0.05	2128.61	987.44	70–13509
PDQ	Low Medium High	25.9 42.3 31.8		3322 (28.9) 4753 (41.4) 3403 (29.6)		
The number of times cooked dinner at home (a week)	Low (0–2) Sometimes (3–5) High (6–7)	13.0 38.3 48.1		1521 (13.4) 3765 (33.2) 6049 (53.4)		

Note. Numbers may not sum to total due to missing data. PDQ = perceived diet quality; SD = standard deviation; SE = standard error. ^a^ Not working = unemployed, retirees, those not actively looking for work.

**Table 3:** Model fit information for LCPA models fit to data.

Class	N. of Parameters	AIC	BIC	Entropy	VLMR ^a^	BLRT ^a^
Daily dietary patterns						
2	34	591,109.562	591,359.409	0.993	*p* < 0.001	*p* < 0.001
3	46	580,090.445	580,428.473	0.996	*p* < 0.001	*p* < 0.001
4	58	575,221.906	575,648.116	0.873	*p* < 0.001	*p* < 0.001
5 ^b^	70	571,428.053	571,942.444	0.867	*p* < 0.001	*p* < 0.001
6	82	569,268.543	569,871.116	0.886	*p* = 0.8711	*p* < 0.001
Dinner dietary patterns						
2	34	540,270.913	540,517.904	0.999	*p* < 0.001	*p* < 0.001
3	46	528,976.751	529,310.916	0.982	*p* < 0.001	*p* < 0.001
4	58	513,747.784	514,169.122	0.999	*p* < 0.001	*p* < 0.001
5	70	512,479.929	512,988.440	0.983	*p* = 0.0107	*p* < 0.001
6 ^c^	82	501,781.884	502,377.569	0.976	*p* < 0.001	*p* < 0.001
7	94	498,611.403	499,294.262	0.971	*p* = 0.5023	*p* < 0.001

Note. AIC = Akaike information criterion; BIC = Bayesian information criterion; BLRT = bootstrapped likelihood ratio test; LCPA = latent class profile analysis; VLMR = Vuong–Lo–Mendell–Rubin likelihood ratio test. ^a^ Chi-square statistic for the VLMR and the BLRT; when non-significant (*p* > 0.05), the VLMR and the BLRT test provide evidence that the K-1 class model fits the data better than the K-class model. ^b^ The 5-class model was selected based on its having a smaller BIC than the 4-class model, and non-significant VLMR in the 6-class model. ^c^ The 6-class model was selected based on its having a smaller BIC than the 5-class model, and non-significant VLMR in the 7-class model.

**Table 4:** Identified LCPA classes from daily and dinner meal calculated HEI-2010 from the NHANES 2007–2010.

	Daily	Dinner
Class 1	SAD	SAD dinner
Class 2	SAD with low sodium	SAD dinner with high sodium
Class 3	Healthy US	SAD dinner with high seafood
Class 4	Healthy US with high vegetable	Healthy US dinner with high vegetable
Class 5	Healthy US with low sodium	Healthy Mediterranean-style dinner
Class 6		Healthy US dinner with low sodium

Note. HEI-2010 = Healthy Eating Index 2010; LCPA = latent class profile analysis; SAD = standard American diet.

**Table 5:** Differences in daily HEI-2010 components and energy (kcal/day) among the latent classes of the NHANES 2007–2010 population (age ≥ 19 years, N = 11,481).

Variables	5-Class Solution					*p* Value
	Class 1 (*n* = 2908, 25%)	Class 2 (*n* = 1568, 14%)	Class 3 (*n* = 3798, 33%)	Class 4 (*n* = 2502, 22%)	Class 5 (*n* = 705, 6%)	
	SAD	SAD with Low Sodium	Healthy US	Healthy US with High Vegetable	Healthy US with Low Sodium	
			Mean (SD)			
Total vegetable	1.87 (1.42)	2.23 (1.73)	3.33 (1.60)	4.09 (1.14)	3.10 (1.40)	<0.001
Green/beans	0.04 (0.18)	0.02 (0.12)	0.04 (0.19)	4.91 (0.28)	2.52 (0.67)	<0.001
Total fruit	1.19 (1.72)	2.32 (2.17)	2.76 (2.14)	2.52 (2.13)	2.31 (2.04)	<0.001
Whole grains	1.12 (2.10)	2.51 (3.31)	2.78 (3.37)	2.48 (3.24)	2.13 (3.01)	<0.001
Dairy	5.00 (3.36)	6.72 (3.35)	4.43 (3.37)	4.66 (3.38)	4.99 (3.25)	<0.001
Total protein foods	4.56 (0.66)	1.68 (0.92)	4.69 (0.61)	4.65 (0.92)	4.55 (1.01)	<0.001
Seafood	1.39 (1.99)	0.83 (1.50)	2.27 (2.29)	2.47 (2.25)	2.66 (2.20)	<0.001
Fatty acid ratio	3.25 (3.08)	3.37 (3.50)	6.46 (3.33)	5.90 (3.50)	5.29 (3.45)	<0.001
Sodium *	5.54 (3.46)	6.17 (3.40)	3.57 (3.41)	3.99 (3.50)	4.26 (3.60)	<0.001
Refined grains *	6.42 (3.50)	4.93 (3.95)	5.73 (3.73)	6.12 (3.73)	5.62 (3.64)	<0.001
SoFAAS *	6.31 (4.35)	9.31 (6.43)	16.27 (3.64)	14.61 (5.50)	12.56 (5.85)	<0.001
Energy (kcal/day)	2323.93 (1132.29)	2037.81 (1057.91)	1908.47 (865.31)	1995.99 (895.34)	2404.42 (1103.41)	<0.001

Note. HEI-2010 = Healthy Eating Index 2010; SAD = standard American diet; SD = standard deviation; SoFAAS = solid fats, alcohol and added sugars. * Lower numerical scores indicate higher consumption.

**Table 6:** Differences in dinner HEI-2010 components and energy (kcal/day) among the latent classes of the NHANES 2007–2010 population (age ≥ 19 years, N = 10,556).

Variables	6-Class Solution						*p* Value
	Class 1 (*n* = 1431, 14%)	Class 2 (*n* = 5028, 48%)	Class 3 (*n* = 1380, 13%)	Class 4 (*n* = 1396, 13%)	Class 5 (*n* = 737, 7%)	Class 6 (*n* = 584, 6%)	
	SAD Dinner	SAD Dinner with High Sodium	SAD Dinner with High Seafood	Healthy US Dinner with High Vegetable	Healthy Mediterranean-Style Dinner	Healthy US Dinner with Low Sodium	
	Mean (SD)						
Total vegetable	1.24 (1.65)	3.17 (1.97)	2.82 (2.03)	4.59 (0.88)	4.48 (0.99)	2.82 (2.33)	< 0.001
Green/beans	0.01 (0.15)	0.03 (0.20)	0.09 (0.38)	4.92 (0.34)	4.89 (0.42)	0.02 (0.20)	< 0.001
Total fruit	0.62 (1.53)	0.69 (1.59)	0.88 (1.71)	0.79 (1.68)	0.80 (1.63)	1.63 (2.26)	< 0.001
Whole grains	0.85 (2.60)	0.65 (2.16)	1.07 (2.81)	0.66 (2.22)	0.92 (2.64)	1.80 (3.68)	< 0.001
Dairy	7.16 (4.05)	3.00 (3.71)	2.67 (3.67)	2.93 (6.64)	2.67 (3.54)	1.81 (3.14)	< 0.001
Total protein foods	0.65 (0.88)	4.76 (0.58)	4.59 (0.93)	4.44 (1.40)	4.88 (0.53)	0.36 (0.66)	< 0.001
Seafood	0.04 (0.27)	0.04 (0.26)	4.83 (0.51)	0.13 (0.44)	4.81 (0.55)	0.04 (0.25)	< 0.001
Fatty acid ratio	1.14 (2.31)	5.00 (3.88)	7.19 (3.79)	5.71 (3.91)	7.13 (3.75)	8.98 (2.36)	< 0.001
Sodium *	4.99 (4.23)	3.30 (3.70)	3.55 (3.91)	2.68 (3.55)	3.23 (3.73)	5.37 (4.30)	< 0.001
Refined grains *	3.94 (4.42)	5.84 (4.10)	5.77 (4.16)	6.36 (4.04)	5.79 (4.19)	5.95 (4.51)	< 0.001
SoFAAS *	7.55 (7.02)	13.41 (6.60)	14.61 (6.46)	15.21 (5.85)	15.89 (5.68)	15.70 (6.73)	< 0.001
Energy (kcal/day)	706.48 (626.33)	850.03 (522.18)	851.57 (563.57)	844.54 (478.80)	914.50 (605.21)	525.48 (474.47)	< 0.001

Note. HEI-2010 = Healthy Eating Index 2010; SAD = standard American diet; SD = standard deviation; SoFAAS = solid fats, alcohol and added sugars. * Lower numerical scores indicate higher consumption.

**Table 7:** Predicting daily dietary patterns (latent classes): multinomial logistic regression of the NHANES 2007–2010 population (age ≥ 19 years, N = 11,481).

Predictors		B (SE)	OR (95% CI)
**Class 2 (SAD with low sodium) vs. Class 1 (*SAD*)**			
Race/ethnicity	Mexican American/Other Hispanic	0.24 (0.10)	1.27 (1.04, 1.54) *
	Non-Hispanic/Black	−0.48 (0.11)	0.62 (0.50, 0.78) ***
	Other	0.47 (0.23)	1.60 (1.01, 2.51) *
	Non-Hispanic/White ^a^		
**Class 3 (Healthy US) vs. Class 1**			
Race/ethnicity	Mexican American/Other Hispanic	0.70 (0.08)	2.01 (1.71, 2.36) ***
	Non-Hispanic/Black	0.13 (0.08)	1.14 (0.97, 1.35)
	Other	1.08 (0.18)	2.95 (2.06, 4.20) ***
	Non-Hispanic/White ^a^		
Educational level	College, graduate or above	0.56 (0.09)	1.75 (1.46, 2.10) ***
	Some college	0.21 (0.07)	1.23 (1.07, 1.42) **
	≤ high school graduate/equivalent ^a^		
Income	High	0.18 (0.08)	1.20 (1.02, 1.39) *
	Middle	0.13 (0.10)	1.13 (0.94, 1.37)
	Low ^a^		
PDQ	High	0.65 (0.09)	1.92 (1.62, 2.27) ***
	Middle	0.36 (0.07)	1.43 (1.24, 1.67) ***
	Low ^a^		
**Class 4 (Healthy US with high vegetable) vs. Class 1**			
Race/ethnicity	Mexican American/Other Hispanic	1.04 (0.09)	2.82 (2.37, 3.36) ***
	Non-Hispanic/Black	0.15 (0.10)	1.16 (0.95, 1.40)
	Other	1.35 (0.19)	3.87 (2.66, 5.63) ***
	Non-Hispanic/White ^a^		
Educational level	College, graduate or above	0.58 (0.10)	1.79 (1.47, 2.18) ***
	Some college	0.13 (0.08)	1.13 (0.96, 1.34)
	≤ high school graduate/equivalent ^a^		
Frequency of cooked dinner at home (a week)	High (6–7)	0.39 (0.15)	1.47 (1.09, 1.98) *
	Moderate (3–5)	0.28 (0.15)	1.33 (0.98, 1.79)
	Low (0–2) ^a^		
PDQ	High	0.86 (0.10)	2.36 (1.96, 2.85) ***
	Middle	0.36 (0.09)	1.44 (1.22, 1.70) ***
	Low ^a^		
**Class 5 (Healthy US with low sodium) vs. Class 1**			
Race/ethnicity	Mexican American/Other Hispanic	0.88 (0.13)	0.41 (1.87, 3.10) ***
	Non-Hispanic/Black	−0.21 (0.16)	0.81 (0.60, 1.11)
	Others	1.32 (0.24)	3.74 (2.32, 6.25) ***
	Non-Hispanic/White ^a^		
Educational level	College, graduate or above	0.83 (0.14)	2.29 (1.73, 3.02) ***
	Some college	0.29 (0.13)	1.34 (1.05, 1.71) *
	≤ high school graduate/equivalent ^a^		

Note. CI = confidence interval; OR = odds ratio; PDQ = perceived diet quality; SAD = standard American diet; SoFAAS = solid fats, alcohol and added sugars. Age, sex/gender, and marital status controlled. Class 1 was used as the reference group. ^a^ The reference category. * *p* < 0.05, ** *p* < 0.01, and *** *p* < 0.001.

**Table 8:** Predicting dinner dietary patterns (latent classes): multinomial logistic regression of the NHANES 2007–2010 population (age ≥ 19 years, N = 10,556).

Predictors		B (SE)	OR (95% CI)
**Class 2 (SAD dinner with high sodium) vs. Class 1 (SAD dinner)**			
Race/ethnicity	Mexican American/Other Hispanic	−0.35 (0.81)	0.70 (0.60, 0.82) ***
	Non-Hispanic/Black	0.53 (0.10)	1.70 (1.40, 2.06) ***
	Other	0.01 (0.19)	1.00 (0.70, 1.45)
	Non-Hispanic/White ^a^		
Frequency of cooked dinner at home (a week)	High (6–7)	0.20 (0.10)	1.23 (1.00, 1.50) *
	Moderate (3–5)	0.17 (0.11)	1.19 (0.97, 1.46)
	Low (0–2) ^a^		
**Class 3 (SAD dinner with high seafood) vs. Class 1**			
Race/ethnicity	Mexican American/Other Hispanic	−0.07 (0.10)	0.93 (0.76, 1.14)
	Non-Hispanic/Black	0.48 (0.12)	1.62 (1.28, 2.06) ***
	Other	0.93 (0.20)	2.54 (1.71, 3.77) ***
	Non-Hispanic/White ^a^		
Educational level	College, graduate or above	0.28 (0.12)	1.32 (1.05, 1.65) *
	Some college	0.19 (0.10)	1.10 (0.92, 1.32)
	≤ high school graduate/equivalent ^a^		
Income	High	−0.30 (0.10)	0.74 (0.61, 0.91) **
	Middle	−0.15 (0.10)	0.87 (0.71, 1.05)
	Low ^a^		
**Class 4 (Healthy US dinner with high vegetable) vs. Class 1**			
Race/ethnicity	Mexican American/Other Hispanic	−0.06 (0.10)	0.94 (0.77, 1.16)
	Non-Hispanic/Black	0.72 (0.12)	2.05 (1.62, 2.59) ***
	Other	0.61 (0.21)	1.84 (1.21, 2.79) **
	Non-Hispanic/White ^a^		
Income	High	−0.31 (0.10)	0.74 (0.60, 0.90) **
	Middle	−0.32 (0.13)	0.72 (0.56, 0.93) *
	Low ^a^		
Frequency of cooked dinner at home (a week)	High (6–7)	0.29 (0.13)	1.34 (1.03, 1.74) *
	Moderate (3–5)	0.34 (0.14)	1.41 (1.08, 1.84) *
	Low (0–2) ^a^		
PDQ	High	−0.48 (0.11)	0.62 (0.50, 0.78) ***
	Middle	−0.18 (0.10)	0.84 (0.69, 1.02)
	Low ^a^		
**Class 5 (Healthy Mediterranean-style dinner) vs. Class 1**			
Race/ethnicity	Mexican American/Other Hispanic	0.18 (0.13)	1.20 (0.94, 1.53)
	Non-Hispanic/Black	0.53 (0.15)	1.69 (1.26, 2.27) ***
	Other	1.29 (0.22)	3.63 (2.37, 5.56) ***
	Non-Hispanic/White ^a^		
Educational level	College, graduate or above	0.47 (0.13)	1.60 (1.23, 2.08) ***
	Some college	0.08 (0.13)	1.08 (0.85, 1.38)
	≤ high school graduate/equivalent ^a^		
Income	High	−0.35 (0.13)	0.71 (0.55, 0.91) **
	Middle	−0.13 (0.15)	0.88 (0.65, 0.18)
	Low ^a^		
Frequency of cooked dinner at home (a week)	High (6–7)	0.45 (0.17)	1.57 (1.13, 2.18) **
	Moderate (3–5)	0.30 (0.17)	1.35 (0.97, 1.90)
	Low (0–2) ^a^		
PDQ	High	−0.42 (0.14)	0.66 (0.51, 0.86) **
	Middle	−0.33 (0.12)	0.72 (0.57, 0.91) **
	Low ^a^		
**Class 6 (Healthy US dinner with low sodium) vs. Class 1**			
Race/ethnicity	Mexican American/Other Hispanic	0.22 (0.13)	1.25 (0.97, 1.62)
	Non-Hispanic/Black	0.37 (0.16)	1.45(1.05, 1.99) *
	Other	1.08 (0.24)	2.94(1.84, 4.72) ***
	Non-Hispanic/White ^a^		

Note. CI = confidence interval; OR = odds ratio; PDQ = perceived diet quality; SAD = standard American diet; SoFAAS = solid fats, alcohol and added sugar. Age, sex/gender, and marital status controlled. Class 1 was used as the reference group. ^a^ The reference category. * *p* < 0.05, ** *p* < 0.01, and *** *p* < 0.001.

## Supplementary Materials

The following are available online at https://www.mdpi.com/2072-6643/12/11/3268/s1, Table S1: Daily HEI components by latent classes of the NHANES 2007–2010 population (age ≥ 19 years, N = 11,481) compared to reported US average scores of HEI-2010 components; Table S2: Dinner HEI components by latent classes of the NHANES 2007–2010 population (age ≥ 19 years, N = 10,556) compared to reported US average scores of HEI-2010 components; Table S3: Prevalence of Daily and Dinner Latent Classes by Gender.
