# Supplementary material for: Cooking Frequency and Perception of Diet among US Adults Are Associated with US Healthy and Healthy Mediterranean-Style Dietary Related Classes: A Latent Class Profile Analysis
Source: Nutrients. 2020 Oct 25;12(11):3268. doi: 10.3390/nu12113268 (PMC7693972; doi:10.3390/nu12113268)
Supplement: Supplementary file 1 [file nutrients-12-03268-s001.pdf]

nutrients MDPI

Supplement Table 1. Daily HEI components by Latent Classes of the 2007-2010 NHANES population (age ≥ 19 years, N=11,481) compared to reported U.S. average scores of HEI-2010 components as reported in Guenther, 2014.

Component		**Class 1**	**Class 2**	**Class 3**	**Class 4**	**Class 5**
	Mean U.S. HEI scores Mean (SE)	SAD	SAD with low sodium	Healthy US	Healthy US with high vegetable	Healthy US with low sodium
Total vegetable	3.2 (0.0)	1.87(1.42)	2.23 (1.73)	3.33 (1.60)	4.09 (1.14)	3.10 (1.40)
Green/beans	1.6 (0.1)	0.04 (0.18)	0.02 (0.12)	0.04 (0.19)	4.91 (0.28)	2.52 (0.67)
Total fruit	2.7 (0.1)	1.19 (1.72)	2.32 (2.17)	2.76 (2.14)	2.52 (2.13)	2.31 (2.04)
Whole grains	1.9 (0.1)	1.12 (2.10)	2.51 (3.31)	2.78 (3.37)	2.48 (3.24)	2.13 (3.01)
Dairy	6.1 (0.1)	5.00 (3.36)	6.72 (3.35)	4.43 (3.37)	4.66 (3.38)	4.99 (3.25)
Total protein	4.5 (0.0)	4.56 (0.66)	1.68 (0.92)	4.69 (0.61)	4.65 (0.92)	4.55 (1.01)
Seafood	3.1 (0.1)	1.39 (1.99)	0.83 (1.50)	2.27 (2.29)	2.47 (2.25)	2.66 (2.20)
Fatty acid	4.3 (0.1)	3.25 (3.08)	3.37 (3.50)	6.46 (3.33)	5.90 (3.50)	5.29 (3.45)
Sodium *	4.9 (0.1)	5.54 (3.46)	6.17 (3.40)	3.57 (3.41)	3.99 (3.50)	4.26 (3.60)
Refined grains *	5.6 (0.2)	6.42 (3.50)	4.93 (3.95)	5.73 (3.73)	6.12 (3.73)	5.62 (3.64)
SoFAAS *	9.4 (0.2)	6.31 (4.35)	9.31 (6.43)	16. 27 (3.61)	14.61 (5.50)	12.56 (5.81)

SAD (Class1,2) vs Healthy US (Class3,4,5)	Low veg<br>Low seafood<br>Low fatty acid<br>Low sodium	High fatty acid<br>High sodium<br>Low SoFAAS

Note: SAD Standard American Diet

* Lower numerical scores indicate higher consumption.

Supplement Table 2. Dinner HEI components by Latent Classes of the 2007-2010 NHANES population (age ≥ 19 years, N=10,556) compared to reported U.S. average scores of HEI-2010 components as reported in Guenther, 2014.

Component		**Class 1**	**Class 2**	**Class 3**	**Class 4**	**Class 5**	**Class 6**
	Mean U.S. HEI scores Mean (SE)	SAD dinner	SAD dinner with high sodium	SAD dinner with high seafood	Healthy US dinner with high vegetable	Healthy Mediterranean style dinner	Healthy US dinner with low sodium
Total vegetable	3.2 (0.0)	1.24 (1.65)	3.17 (1.97)	2.82 (2.03)	4.59 (0.88)	4.48 (0.99)	2.82 (2.33)
Green/beans	1.6 (0.1)	0.01 (0.15)	0.03 (0.20)	0.09 (0.38)	4.92 (0.34)	4.89 (0.42)	0.02 (0.20)
Total fruit	2.7 (0.1)	0.62 (1.53)	0.69 (1.59)	0.88 (1.71)	0.79 (1.68)	0.80 (1.63)	1.63 (2.26)
Whole grains	1.9 (0.1)	0.85 (2.60)	0.65 (2.16)	1.07 (2.81)	0.66 (2.22)	0.92 (2.64)	1.80 (3.68)
Dairy	6.1 (0.1)	7.16 (4.05)	3.00 (3.71)	2.67 (3.67)	2.93 (6.64)	2.67 (3.54)	1.81 (3.14)
Total protein	4.5 (0.0)	0.65 (0.88)	4.76 (0.56)	4.59 (0.93)	4.44 (1.40)	4.88 (0.53)	0.36 (0.66)
Seafood	3.1 (0.1)	0.04 (0.27)	0.04 (0.26)	4.83 (0.51)	0.13 (0.44)	4.81 (0.55)	0.04 (0.25)
Fatty acid	4.3 (0.1)	1.14 (2.31)	5.00 (3.88)	7.19 (3.79)	5.71 (3.91)	7.13 (3.75)	8.98 (2.36)
Sodium *	4.9 (0.1)	4.99 (4.23)	3.30 (3.70)	3.55 (3.91)	2.68 (3.55)	3.23 (3.73)	5.37 (4.30)
Refined grains*	5.6 (0.2)	3.94 (4.42)	5.84 (4.10)	5.77 (4.16)	6.36 (4.04)	5.79 (4.19)	5.95 (4.51)
SoFAAS *	9.4 (0.2)	7.55 (7.02)	13.41(6.60)	14.61 (6.46)	15.21 (5.85)	15.89 (5.68)	15.70 (6.73)

SAD (Class 1,2,3) vs Healthy US or Med Style (Class 4,5,6)	Low veg<br>Low seafood<br>Low fatty acid<br>Low sodium	High fatty acid<br>Low SoFAAS

Note: SAD Standard American Diet

* Lower numerical scores indicate higher consumption.

Supplement Table 3. Prevalence of Daily and Dinner Latent Classes by Gender

	n (%)		***p* value**
	**Female** (n = 5058)	**Male** (n = 5623)	
**Daily latent classes**			
Class 1 (SAD)	1296 (22.1)	1612 (28.6)	< .001
Class 2 (SAD with low sodium)	896 (15.3)	672 (12.0)	
Class 3 (Healthy US)	1958 (33.4)	1840 (32.7)	
Class 4 (Healthy US with high vegetable)	1339 (22.9)	1163 (20.7)	
Class 5 (Healthy US with low sodium)	369 (6.3)	336 (6.0)	
**Dinner latent classes**			
Class 1 (SAD dinner)	762 (14.1)	669 (13.0)	< .001
Class 2 (SAD dinner with high sodium)	2471 (45.7)	2557 (49.6)	
Class 3 (SAD dinner with high seafood)	710 (13.1)	670 (13.0)	
Class 4 (Healthy US dinner with high vegetable)	755 (14.0)	641 (12.4)	
Class 5 (Healthy Mediterranean style dinner)	374 (6.9)	363 (7.0)	
Class 6 (Healthy US dinner with low sodium)	333 (6.2)	251 (5.0)	

Note: SAD Standard American Diet
